# Larger brains and relatively smaller cerebella in Asian elephants compared with African savanna elephants

**DOI:** 10.1093/pnasnexus/pgaf141

**Published:** 2025-05-20

**Authors:** Malav Shah, Olivia Heise, Peter Buss, Lin-Mari de Klerk-Lorist, Stefan Hetzer, John-Dylan Haynes, Thomas Hildebrandt, Michael Brecht

**Affiliations:** Bernstein Center for Computational Neuroscience Berlin, Humboldt-Universität zu Berlin, Philippstr. 13, Haus 6, 10115 Berlin, Germany; Bernstein Center for Computational Neuroscience Berlin, Humboldt-Universität zu Berlin, Philippstr. 13, Haus 6, 10115 Berlin, Germany; Veterinary Wildlife Services, Kruger National Park, South African National Parks, Skukuza 01350, South Africa; South African Department of Agriculture, Land Reform and Rural Development (DALRRD), Skukuza, Mpumalanga 01350, South Africa; Berlin Center for Advanced Neuroimaging (BCAN), Bernstein Center for Computational Neuroscience Berlin, Charité—Universitätsmedizin Berlin, Sauerbruchweg 4, Charitéplatz 1, Berlin 10117, Germany; Berlin Center for Advanced Neuroimaging (BCAN), Bernstein Center for Computational Neuroscience Berlin, Charité—Universitätsmedizin Berlin, Sauerbruchweg 4, Charitéplatz 1, Berlin 10117, Germany; Leibniz Institute for Zoo and Wildlife Research, Alfred-Kowalke-Strasse 17, D-10315 Berlin, Germany; Bernstein Center for Computational Neuroscience Berlin, Humboldt-Universität zu Berlin, Philippstr. 13, Haus 6, 10115 Berlin, Germany; NeuroCure Cluster of Excellence, Humboldt-Universität zu Berlin, Berlin 10117, Germany

**Keywords:** brain size, African elephant, Asian elephant, brain growth, brain evolution

## Abstract

Elephants are the largest terrestrial animals, but our knowledge of their brains is limited. We studied brain size, proportions, and development in Asian (*Elephas maximus*) and African savanna (*Loxodonta africana*) elephants. Specifically, we weighed, photographed, and analyzed postmortem magnetic resonance scans of elephant brains in addition to collecting elephant brain data from the literature. Despite their smaller body size, adult Asian female elephants have substantially and significantly heavier brains (mean 5,346 ± 916 g SD) than adult African savanna female elephants (mean 4,417 ± 593 g SD). In line with their larger body size, adult African savanna male elephants (mean 5,603 ± 1,159 g SD) have significantly heavier brains than African female elephants; the brain weight of the adult male Asian elephant remains unclear. Elephant brain weight increases ∼3-fold postnatally. This postnatal increase is similar to that of the human brain but is larger than that seen in nonhuman primates. Asian elephants likely have more cerebral cortical gray matter than African ones; their cerebellum is relatively smaller (19.1% of total brain weight) than in African elephants (22.3%). Our data indicate a higher degree of encephalization in Asian than in African savanna elephants. The massive postnatal brain growth of elephants is likely related to prolonged adolescence and the important role of experience in elephant life history.

**Significance Statement**

Elephants are fascinating animals. Despite such fascination, our knowledge of the elephant brain is limited, and neuroanatomical differences between Asian and African elephants are largely unexplored. We collected a large number of elephant brains and studied their macro-anatomy to characterize species differences. To our surprise, we found that Asian elephants have larger brains and larger gray-matter volume than African elephants, and this brain size difference contrasts with the smaller bodies of Asian elephants. We also find that Asian elephants have relatively (in proportion to the whole brain size) smaller cerebella. Elephant brains grow thrice postnatally—an exceptionally large postnatal brain growth.

## Introduction

Morphological differences between elephant species are widely known. For example, African savanna elephants (*Loxodonta africana*) are larger and have larger ears compared with Asian elephants (*Elephas maximus*); only African elephant cows are known to develop large tusks compared with rudimentary tusks in Asian cows. These differences reflect the substantial genetic separation of Asian and African savanna elephants, which is suggested to date back 5–8 million years (1–3). Further, Racine (4) suggests that trunk-related morphological difference—the presence of two trunk fingers in African elephants compared with one trunk finger in Asian elephants—in these two species belonging to two separate genera results in behavioral differences, e.g. African elephants frequently use a two-fingered pinch to grasp objects, while Asian elephants wrap their trunks around objects (5, 6).

Interestingly, elephant species also appear to differ in their human interactions. Asian elephants, in particular, have a long history of semi-domestication (7) and of being used as a workforce by humans in numerous cultures and locations (8, 9). African forest elephants have also been used by humans; however, the semi-domestication of African forest or savanna elephants is more of an exception than the rule. An attempt by the Belgian king Leopold to establish an “Asian-like” work use of African forest elephants instructed by Indian mahouts met with partial success (9). Therefore, it is generally thought that African elephants are more difficult to habituate than Asian elephants (8–12).

Social factors may also shape elephant brains. Elephants live in complex social groups. Experience plays a major role in shaping these fusion–fissure societies (13), and according to the social brain hypothesis (14), social complexity might be a driving factor in elephant brain evolution.

Despite well-known behavioral differences, we are largely ignorant about brain differences of elephant species. This ignorance, however, reflects the inaccessibility of elephant brains to a large extent. Most early scientific reports on elephant brains have used single specimens extracted from zoo elephants ((15), reviewed in (16)). This format is clearly not suitable for detecting species differences. The study by Shoshani and colleagues (17) proved to be a turning point in the field of elephant brain investigation, which not only provided a detailed description of brain structures but was also based on multiple elephant brains (six complete brains with full documentation) and a large-scale review of elephant brain data (identifying 15 complete elephant brains with partial documentation). This database, in addition to pooled brain data, allowed Shoshani and colleagues to report across-species averages. Here, we follow in Shoshani and colleagues’ footsteps and combine their earlier data with a several-decade elephant brain collection effort from our side. Thus, we had access to an additional 19 complete elephant brains with full documentation, and we also analyzed a subset of these brains with magnetic resonance (MR) imaging.

With access to this dataset, we ask the following questions: (i) How does elephant brain weight differ across species and sexes? (ii) What is the postnatal weight increase of the elephant brain? (iii) How much cortical gray matter (GM) do elephants have? (iv) What is the relative and absolute size of the elephant cerebellum in elephant species?

## Results

We compiled brain weight data from Asian (*E. maximus*) and African savanna (*L. africana*) elephants; we had no access to African forest (*Loxodonta cyclotis*) elephant brains. Brain weight measurements came from three sources: (i) elephant brains extracted by us from zoo elephants (*n* = 14) or from wild elephants (*n* = 5), (ii) elephant brains extracted by Shoshani and colleagues and described in (17) (*n* = 6), and (iii) elephant brain weights we extracted from the literature. The brain data (with sources) are provided in Table 1. Kindly note that elephant brain extraction is not trivial (5, 34), and the quality of the samples included here varies greatly.

**Table 1.** Overview of elephant and brain data.

Species, name	Ref.	Sex	C/W	Age (years)	Last location	Body weight (t)	Brain weight (g)	Cerebellum (%)
*L. a.*, Zimba	(0)	F	C	39	Opel-Zoo Kroneberg, DE	—	4,740	—
*L. a.*, Indra	(0)	F	C	35	Elefantenhof Platschow, DE	2.75	3,907	19.92^v^
*L. a.*, Linda	(0)	F	C	35	Zoo Poznan, PL	—	5,020	—
*L. a.,* Aruba	(0)	F	C	41	Opel-Zoo Kroneberg, DE	—	3,730	30.29^w^
*L. a.,* Bibi	(0)	F	C	41	Zoo Aalborg, DK	3.320	4,310	25.29^w^
*L. a.,* Timba	(0)	F	C	45	Elefantenhof Platschow, DE	—	4,490	—
*L. a.,* Tanja	(0)	F	C	42	Zoo Aalborg, DK	4.040	6,195	18.53^v^
*L. a.,* Nancy	(17)	F	C	46	Zoo Washington, DC, US	3.505	4,420	16.63^w^
*L. a.,* Kenya	(17)	F	C	24	Kountze Texas, US	1.7933	4,050	—
*L. a.,* –	(18)	F?	W	Ad	—	2.75	4,480	−^?^
*L. a.,* –	(19)	F	W	Ad	—	2.16	4,100	—
*L. a.,* –	(19)	F	W	Ad	—	2.537	4,000	—
*L. a.,* –	(20)	F	C	25?	—	—	4,027	23.75^v^
*L. a.,* –	(21)	F	C	Ad.	Amsterdam	1.642	4,370	—
*L. a.,* –	(0)	M	W	22	Kruger NP, SA	—	6,700	—
*L. a.,* –	(0)	M	W	28	Kruger NP, SA	—	5,700	—
*L. a.,* –	(0)	M	W	22	Kruger NP, SA	—	4,900	—
*L. a.,* –	(0)	M	W	27	Kruger NP, SA	—	5,000	—
*L. a.,* –	(0)	M	W	39	Kruger NP, SA	—	5,300	—
*L. a.,* –	(22)	M	W	30	Zambia	4.3801	9,000	—
*L. a.,* –	(23)	M	W	Ad	**—**	—	6,000	—
*L. a.,* –	(24)	M	W	Ad	Maji Moto Camp, Africa	6.654	5,712	—
*L. a.,* –	(19)	M	W	Ad	East Africa	5.55	5,300	—
*L. a.,* –	(22)	M	W	40	Zambia	5.1744	4,000	—
*L. a.,* LA1	(25)	M	W	20–30	Malilangwe Trust, Chiredzi, ZW	—	5,145	21.23^v^
*L. a.,* LA2	(25)	M	W	20–30	Malilangwe Trust, Chiredzi, ZW	—	5,250	22.46^v^
*L. a.,* LA3	(25)	M	W	20–30	Malilangwe Trust, Chiredzi, ZW	—	4,835	22.99^v^
*L. a.,* –	(26)	?	C?	Ad	**—**	4	4,210	25.1^v^
*L. a.,* AM1	(0)	M	C	0	Colchester Zoo, UK	0.160	1,759	—
*L. a.,* –	(17)	M	C	0	Zoo Toledo, Ohio, US	0.159	1,724	19^w^
*L. a.,* –	(18)	?	W	0	**—**	0.12	1,650	—
*E. m.,* Dumba	(0)	F	C	44	Elefantenhof Platschow, DE	—	4,840	16.87^v^
*E. m.,* Burma	(0)	F	C	52	Zoo Augsburg, DE	—	4,950	24.44^w^
*E. m.,* Zinda	(0)	F	C	8	Dublin Zoo, UK	—	4,370	—
*E. m.,* Tulsa	(17)	F	C	34	Seagoville Texas, US	3.216	5,220	18.39^w^
*E. m.,* Missy	(17)	F	C	33	Zoo Detroit, Michigan, US	3.4504	5,000	20^w^
*E. m.,* Iki	(17)	F	C	46	RBBB Circus, US	2.2674	4,550	18.86^w^
*E. m.,* –	(27)	F	C	Ad.	Calcutta, IN	0.19754	7,475	—
*E. m.,* Alice	(28)	F	C	50	Luna Park, NYC, US	3.1901	6,075	—
*E. m.,* –	(21)	F	?	25	**—**	2.047	4,660	—
*E. m.,* –	(29)	M	C	22	Yorkshire, UK	3.048	5,443	—
*E. m.,* Luka	(0)	M	C	49	Zoo Osnabrück, DE	4.140	6,065	17.6^w^
*E. m.,* –	(30)	?	?	Ad	**—**	2.547	4,603	—
*E. m.,* –	(31)	?	?	Ad?	**—**	—	5,430	20^w^
*E. m.,* –	(32)	?	?	Ad?	**—**	3.048	4,717	—
*E. m.,* –	(32)	?	?	Ad?	**—**	2.047	4,048	—
*E. m.,* A.B.	(0)	F?	C	0	Hagenbeck, DE	0.125	1,860	17.36^v^
*E. m.,* Raj	(0)	M	C	4	Hagenbeck, DE	0.1	4,350	18.73^v^
*E. m.,* –	(33)	?	C	0.66	**—**	—	2,986^a^	—
*E. m.,* –	(21)	F	?	?	**—**	0.468	3,756	—
								
—	(16)	?	C	Ad	**—**	—	6,500	—

Animals are listed according to species, adults (female, male, unknown sex), juveniles, unknown species. We included all brain weights known to us with exception of the newborn brain weights from (19), which we excluded because of contradictory weight values in Sikes’ book. The exclusion of the newborn brain weights from (19) also explains the differences between our and Shoshani's newborn elephant brain weight estimates.

*L. a.*, *Loxodonta africana*; *E. m.*, *Elephas maximus*; C, captive; W, wild; v, measured using volume; w, measured using weight; (0), data from our study, ?, no coclusive information available.

^a^400 g of assumed dura weight subtracted.

**Fig. 1.**
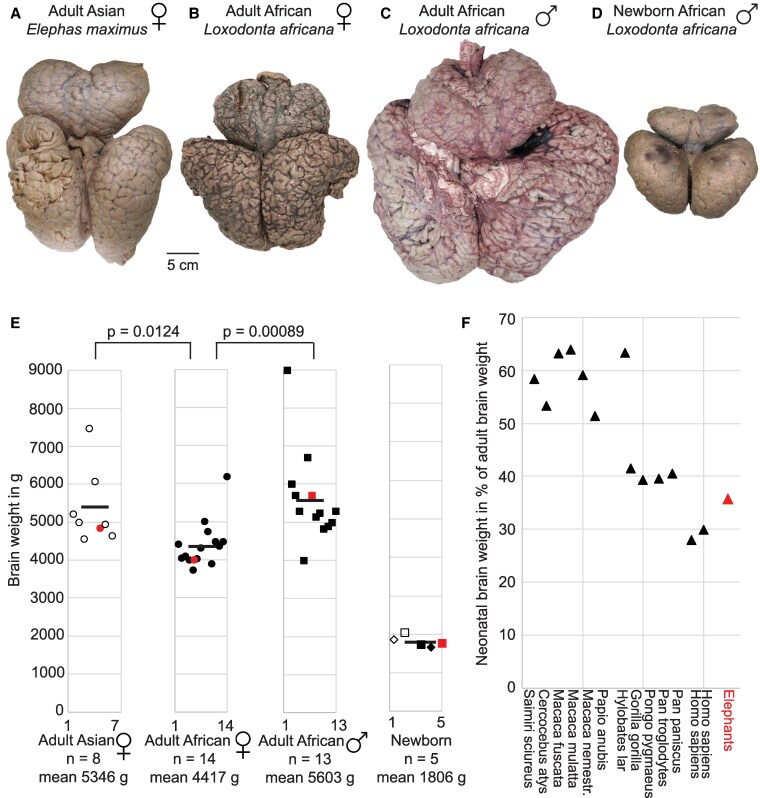
Elephant brain size differs between species and sexes and shows a comparatively large postnatal increase. A) Top view of the brain of a 44-y-old Asian female elephant, Dumba. B) Top view of the brain of a 35-y-old African female elephant, Indra. C) Top view of the brain of an ∼28-y-old feral African male elephant; aging was performed according to tooth abrasion patterns. D) Top view of the brain of a newborn African male elephant. E) Brain weights for adult Asian (*E. maximus*), adult female African (*L. africana*), adult male African (*L. africana*) elephants and brain weights for newborn Asian (empty symbol, *E. maximus*) and African (filled symbols, *L. africana*) elephants. Squares (males), circles (females) and rhombuses (unknown sex). The average brain weight of newborns (1,806 g) is a third (35.4%) of the brute force average brain weight of adult Asian and African elephants (5,107 g). Data are lacking for adult Asian male elephants. *P*-values refer to t-tests, and lines indicate means. Red symbols refer to brain weights of specimens shown in A–D. F) Postnatal brain weight increase in primates and elephants. Relative (to adult brain weights) neonatal brain weights are plotted on the *y*-axis, and smaller values indicate larger postnatal increase. Primate data are replotted from (35) according to nonhuman primate data from Yerkes NPRC and others and two human datasets (36, 37).

We show brain samples and weights of adult and newborn elephants in Fig. 1. In our own data, we referred to elephants >10 years as adults; data from literature were included for only adult animals whose age and sex information was reported. A visual inspection of elephant brain size across species and sex (Fig. 1, top panel) indicates brain size differences across species and sex. A photograph of the brain of an Asian female elephant, Dumba (44 years old), is shown in Fig. 1A. Dumba's brain is larger than Indra, a 35-y-old female African elephant's brain (Fig. 1B). A very large brain of an adult male African elephant is shown in Fig. 1C. The brain of newborn elephants is smaller compared with adult brains, as shown in Fig. 1D for a newborn African male elephant.

Statistics on adult elephant brain weights (Fig. 1E) confirm the impression from these sample brains. Female Asian elephants—albeit their body weight is on average 10–15% smaller than African females (38, 39)—have significantly (two-sided t test, *P* = 0.0124) heavier brains than African female elephants. This brain weight difference is substantial (0.94 kg; 21% of the African female elephant brain weight). We also find that—in line with the well-known body weight differences—adult African male elephants have heavier (*P* = 0.00089) brains than adult African female elephants (Fig. 1E). To our knowledge, there is only one (5,443 g) brain weight of presumably an adult Asian male elephant reported in the literature (29). We extracted a 4,350 g brain from Raj (4 year old), clearly a subadult male Asian elephant, and a large 6,065 g brain from Luka, an adult male Asian elephant bull. We also analyzed body weight–brain weight relationships and encephalization quotients (EQs) and show these results in Fig. S1. However, the strength of the conclusions was limited here by the low number of animals for which both body and brain weight were available.

We also studied newborn elephants’ brain weights (Fig. 1F). Brain weights of newborn elephants showed little scatter and averaged 1,806 g, when pooling across Asian and African elephants. This is approximately three times (35.4%) smaller than the brute force average across adult elephant brain weights (5,107 g). The smaller scatter of the newborn elephant brain weights might be related to the much easier and more reproducible extraction in newborns compared with adult elephants. A comparison of postnatal brain growth in elephants with a few selected primate species (compiled by (35)) is shown in Fig. 1F. In monkeys and gibbons relative neonatal brain weights range between 65 and 50% of adult brain weights (Fig. 1F, left), around 40% in apes, and around 20% in humans (Fig. 1F, middle). Elephants’ relative neonatal brain weights (red in Fig. 1F) appear to fall between humans and nonhuman primates. We conclude that there are marked sex and species differences in elephant brain weight, and the elephant brain growth is large compared with most of the mammals. We also explored the relationship of age vs. brain weight (Fig. S2); the data indicate increasing brain weight up to around the age of 10 years and little systematic change of brain weights beyond 20 years of age.

**Fig. 2.**
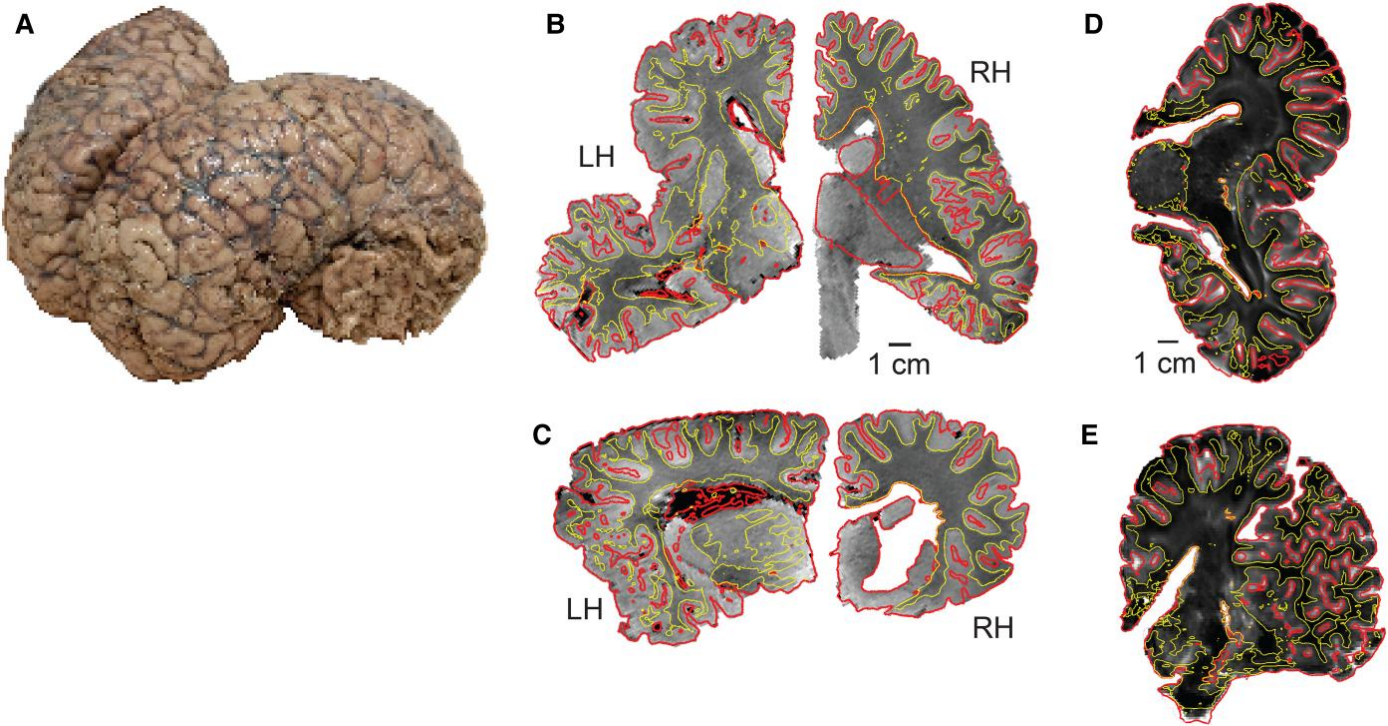
Segmentation of elephant brains using MR scans. A) A left-hand side view of Indra (a 35-y-old female African elephant) as a sample brain. B) A sample horizontal section of T1-weighted MR scan of Indra's brain. Yellow curves show boundaries between WM and GM as a surface generated after segmentation, whereas red curves show GM and pial matter boundaries in B–E. C) A coronal slice from a (structural) T1-weighted MR scan of Indra's brain; conventions as in B. D) A horizontal slice from a (structural) T2-weighted MR scan of the left hemisphere of Dumba (a 44-y-old Asian female elephant); conventions as in B. E) A coronal slice from a (structural) T2-weighted MR scan of Dumba's (a 44-y-old Asian female Elephant) left hemisphere; conventions as in B.

We studied elephant brain macro-anatomy, using volume- and surface-rendering techniques on MRI scans. We describe this methodology in Fig. 2. We acquired multiple T1- and T2-weighted MR image series of Asian and African elephant brains. The combined analyses of T1- or T2-weighted MR images made our analysis more robust against various artifacts. We segmented MR volumes for the best possible rendering of white matter (WM), GM, and pial surface (Pial). Brain volumes were segmented using a variety of image analysis tools (see Materials and methods). The output of this segmentation was a label volume, with each voxel explicitly having one of the three labels—WM, GM, or Pial. The segmentation output is shown for the brain of an African female elephant Indra (Fig. 2A); its T1-weighted MRI slices are shown in Fig. 2B (horizontal plane) and Fig. 2C (coronal plane). The red and yellow curves show Pial–GM segmentation and WM–GM segmentation boundaries, respectively. Both these boundaries were tessellated and further smoothed (see Materials and methods). Figure 2D and E shows T2-weighted MR images from mutually normal planes from the brain of Dumba—a female Asian elephant. The red and yellow curves show again Pial–GM and WM–GM boundaries. MR scans were not only used to determine cerebral GM and WM volumes but also to segment and determine cerebellar volumes. We conclude that MR scans effectively provide quantitative means to segment large elephant brains.

**Fig. 3.**
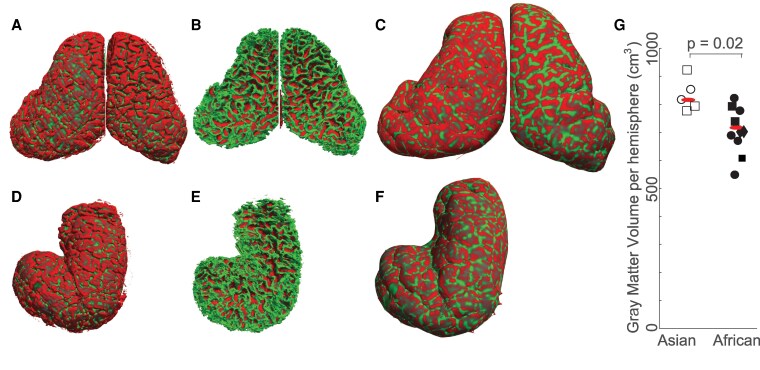
Cerebral cortex shape and GM in African and Asian elephant brains. A) 3D reconstruction generated for the outermost cortical surface (see the red curve in Fig. 2B and C for methodology) for Indra (a 35-y-old female African elephant). B) WM–GM boundary surface for Indra (yellow curve in Fig. 2). C) Inflated version of the cortical surface of Indra as in A, where the green and red colors indicate deep sulci and uppermost gyri, respectively. D) 3D reconstruction generated for the outermost cortical surface of the left hemisphere of Asian female elephant Dumba (a 44-y-old elephant). E) WM–GM boundary surface for Dumba. F) Inflated version of the cortical surface of Dumba. G) GM volume within individual hemispheres of Asian and African elephants; small red horizontal lines indicate means in respective columns; circles and squares indicate female and male adults, respectively. The GM volumes provided here include subcortical structures and hence are larger than exclusively cortical matter volumes. The *P*-value refers to an unpaired t-test. Adults with unknown sex are indicated with rhombuses.

Our segmentation enabled us to generate the cortical GM–Pial boundary, i.e. to visualize the 3D brain surface. We show this surface for the brain of the African female elephant, Indra, in Fig. 3A. The binary color code of these surfaces is based on their curvature; i.e. if a surface node belongs to a surface segment having a certain curvature, it is labeled with “curved” (green), otherwise “flat” (red) value. Therefore, we see a large portion of the brain surface as “flat”—representing gyri, and a tiny portion of “curved” sulci buried deep but visible through gyri gaps in Fig. 3A. Following the same methodology, we also generated the cortical WM–GM boundary as a surface in 3D (the WM surface), and show it for Indra's brain in Fig. 3B. We use the same color coding of curvature as before, revealing a large “curved” surface portion, indicating WM related to gyral peninsulas, and a small portion of “flat” surface, indicating WM related to sulci in Fig. 3B. Figure 3C shows the inflated brain surface of Indra's brain, with gyri (coded in red) and sulci (coded in green). The same segmentation in brain surface (Fig. 3D), WM surface (Fig. 3E), and inflated brain surface (Fig. 3F) is shown for the brain of Asian female elephant Dumba; we restricted the analysis to the left hemisphere in this case, as there was damage to the right hemisphere.

Determination of these surfaces enabled us to estimate GM volume for individual hemispheres by integrating surface area with the normal distance between cortical and WM surfaces. We found that GM volume in Asian elephant brains (832 ± 23 cm^3^, mean **±** SE, average over GM volume per hemisphere) is significantly (two-sided t test = 0.02) larger than in African elephant brains (721 ± 21 cm^3^). The volumetric analysis of elephant brains reveals their gyrification, and based on the statistical test, we conclude that Asian elephants have larger cerebral GM volume than African elephants.

**Fig. 4.**
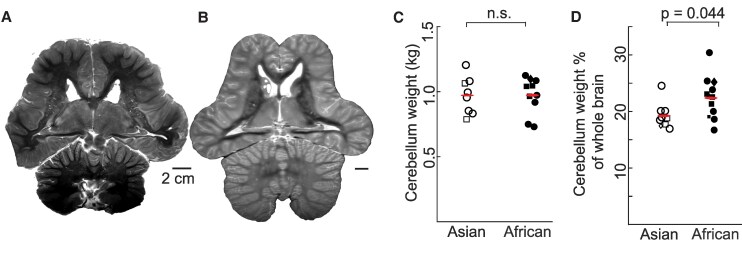
African elephants have proportionally larger cerebella than Asian ones. A) A horizontal slice from T2-weighted MR scan of a newborn Asian baby elephant. B) A horizontal slice from T1-weighted MR scan of an adult male African elephant. Note the large cerebellum (lower part of the scan). C) Absolute cerebellum weights of adult Asian and African elephant brains; the weights are not different between the two species. The *P*-value refers to a t-test. D) Relative cerebellum weights (with respect to whole-brain weight) of Asian and African elephants; relative weights include data from newborn animals and are different between the two species. The *P*-value refers to a t-test. For C and D, small red horizontal lines indicate means in respective columns; circles and squares indicate female and male adults, respectively. Adults with unknown sex are indicated with rhombuses. Smaller symbols indicate newborn babies, conventions as for adults.

Elephants have the largest cerebellum relative to all other mammals (25, 40). We wondered whether cerebellum size differed between African and Asian elephants. Visual inspection of MR scans indeed showed a relatively smaller cerebellum in an Asian stillborn baby elephant brain (Fig. 4A) than in an adult African elephant brain (Fig. 4B). We then assembled absolute and relative cerebellum size measures from (i) our elephant brain MR scans, (ii) elephant brains we had dissected, and (iii) literature reports. We found that absolute cerebella weights were very similar for adult Asian (977 ± 54 g SE) and adult African (978 ± 46 g SE) elephants (Fig. 4C; two-sided t-test, *P* = 0.98). As shown in Fig. 4D, however, relative cerebellum size (percentage of total brain weight) was significantly larger in African elephants (22.29 ± 1.15% SE) than in Asian elephants (19.14 ± 2.26%; two-sided t-test, *P* = 0.044). We conclude that African elephants have a relatively larger cerebellum than Asian elephants.

## Discussion

### Summary

We studied brain weight, proportions, and development in Asian and African elephants. We find that Asian female elephants have heavier brains than African female elephants. African male elephants also have heavier brains than African female elephants. Elephants show a 3-fold postnatal brain weight increase, larger than observed in nonhuman primates. African elephants have relatively larger cerebella than Asian elephants. Furthermore, the data also indicate a trend towards a larger cerebral GM volume in Asian elephants than in African ones.

### Brain proportions of elephants

Our findings on the brain proportions of elephants agree with earlier work describing these brains (17, 20, 34, 41). Our sample size of elephant brains is still limited but allows for a number of conclusions nonetheless. We observe large brains with particularly large forebrains. The smaller brain size of African elephants explains why Asian elephants come out with significantly larger (*P* = 0.02) GM volume than African elephants.

### Species and sex differences in brain weight

Our discovery that Asian female elephants have heavier brains than African female elephants is remarkable in several aspects. First, it is surprising that we only learn about such differences now, despite living in close contact with elephants for thousands of years. Secondly, the low *P*-value reflects that the difference is rather clear. Moreover, the difference is not marginal but prominent (0.94 kg **=** 21% of the female African elephant brain weight). Fourth, the heavier brains in Asian female elephants are unexpected given the larger size of African elephants (38, 39). Thus, our data suggest that Asian elephants have a higher degree of encephalization than their African counterparts. We wonder whether the differences in brain size are related to differences in social structure between the two species, an issue on which only a little information is available. We also speculate that the larger brain size of Asian elephants compared with African savanna elephants might underlie the apparent greater ease of taming/semi-domestication of these animals (8–12). At least in captivity, Asian elephants appear to reach older ages than African savanna elephants (42). We therefore wonder whether the larger brain size of Asian elephants compared with African elephants is related to a stronger reliance on experience-dependent adjustment of behavior in Asian elephants.

We also find that adult male African elephants have substantially (by 1.2 kg) larger brains than adult female African elephants. We reckon that such differences reflect the larger body size of male African elephants. Encephalization coefficients’ estimation according to (31), when adjusted for body size, most male animals indeed have lower EQ values than females (17). We note, however, that one of the male elephant brain weights goes very high and was excluded as an outlier by Shoshani and colleagues (17). The overall impression that the heavier brains of African male elephants compared with female elephants are related to body size is supported by body weight vs. brain weight analysis shown in Fig. S1.

### Postnatal increase of the elephant brain

We observe an approximately three fold postnatal increase in elephant brains. This value corroborates an earlier estimate of postnatal brain growth for African elephants by (43). Our estimate of brain growth deviates from the lower estimate by (17). The reasons for this discrepancy are unclear. We looked into the references by Shoshani and colleagues, and confirmed most of the newborn brain weights, except from (19), which contained conflicting values regarding newborn baby brains; we therefore disregarded them. Our conclusions on the slow postnatal growth of the elephant brain also agree with the morphological developmental research work from (44–46). The approximate three fold postnatal increase of the elephant brain is an unusual postnatal growth for a mammalian brain. Only humans show similar postnatal growth (35) among primates. In humans, the small brain size at birth is associated with a narrow birth channel due to the pelvic bone. Given the large size of the elephant baby’s head, a similar explanation might apply. Alternatively, the small brain size at birth might be related to postnatal learning, as discussed. We note that elephants give birth to precocial offspring after a very long (22 months) gestation. We would expect that such a pattern of extended gestation (giving birth to more advanced newborns) should decrease postnatal growth. To put it into a human-centric perspective, a 13-month-old human infant (the same age as a newborn elephant) has a brain weight of about 850 g, and the brain grows only about 37% to adulthood. The most intuitive explanation for this large postnatal increase in elephant brain size is the major role of experience in the life history of elephants. It appears that experience—particularly the knowledge carried by elephant matriarchs—is indeed a key determinant of elephant group behavior (13). Laws (43) also pointed out that elephant longevity, prolonged adolescence, and high parental investment in a limited number of calves per cow are related to the slow-growing brain and reliance on intelligence. Studies on captive elephants further indicate that even successful birth and bringing up of newborn elephants is experience dependent in elephants (5, 47).

### What might explain the larger cerebellum in African elephants compared with Asian elephants?

Both Asian and African elephants have a very large cerebellum, which also contains the vast majority (97.5%) of neurons in the elephant brain (41). Allometric analysis shows that elephants have relatively the largest cerebellum of all mammals (25). We further find that African elephants have a relatively larger (by ∼15%) cerebellum than Asian elephants. We wonder whether the overall large cerebellum size in both Asian and African elephants and the even larger relative size in African elephants is at least in part related to the elephant trunk and its delicate maneuverability (48), as well as its ability to pick up vibrations (48), and information on texture, size, shape, and temperature is ascertained (49). Particularly, the morphological trunk-tip differences responsible for behavioral differences like “pinching” vs. “wrapping” in African vs. Asian elephants might contribute to size and complexity differences in cerebella.

The trunk is the key morphological specialization of elephants. The large size of the trigeminal ganglion, whose maxillary branch innervates the trunk via the infraorbital nerve, weighing almost as much as a monkey brain, gives a glimpse of an outstanding sensory-motor neuronal information processing of the elephant trunk (50). The elephant trunk is of incredible muscular complexity (5, 51), containing about 90,000 independent muscle fascicles in Asian elephants (52). The trunk-related neural structures differ between Asian and African elephants. Specifically, the facial nucleus, a trunk-related motor control structure (53), contains about 14% more neurons in African than in Asian elephants. The putative trunk module of the trigeminal nucleus, a trunk-related sensory structure (54), also contains about 14% more neurons in African than in Asian elephants. Note that the larger size of trunk-related structures is unexpected given their overall smaller brain. Thus, neural allometry is consistent with the role of the trunk in driving elephant cerebellum extension.

## Conclusion

Our data indicate that elephant brains differ more markedly between elephant species and sexes than previously known. Understanding such brain differences and the extraordinary postnatal growth of the elephant brain will elucidate elephant biology, cerebellar function, and the architecture of these large brains.

## Materials and methods

### Elephant brain data

Our article includes two types of data from elephant brains: brain weight measurements and brain proportion measurements using MR scans. Brain weight measurements came from three kinds of sources: (i) elephant brains we extracted from Zoo animals (*n* = 14) or wild elephants (*n* = 8; for three of which Paul Manger kindly provided data and MR scans); a subset of these specimens was also analyzed by MR scans as detailed below; (ii) elephant brains extracted by Shoshani et al. ((17); *n* = 6); and (iii) elephant brain data we extracted from the literature. Aspects of our methods were described in detail in our recent publications (50, 53) and we only repeat key aspects here.

### Elephant specimens and brains, we extracted

We worked with elephant specimens from two sources. First, most specimens were zoo animals and were collected by the Leibniz Institute for Zoo and Wildlife Research (IZW, Berlin) over the last three decades in agreement with Convention on International Trade in Endangered Species of Wild Fauna and Flora (CITES) regulations. Specifically, specimen reports and CITES documentation for all animals included are held at the IZW. All animals included in the study died of natural causes or were euthanized by experienced zoo veterinarians for humanitarian reasons, because of insurmountable health complications. In most adult elephants, heads were removed at respective zoos, and the remaining skulls were stripped with motorized saws and axes at the Leibniz-IZW Berlin. Some of the brains from skulls were extracted by Franscisca Egelhofer and Anistan Sebastianpillai at the Neuropathology Department Charité Berlin. Secondly, another subset of animals was wild African male elephants (*n* = 5). None of these animals were killed for the purpose of this study either. Five of the brains came from damage-causing animals in Kruger National Park, and the brains were collected at the Kruger National Park under the South African government permit. The age of these animals was determined by tooth replacement patterns as detailed in (43). The rest of the three male adult elephant (LA1/2/3) brains were collected by the Manger group; their information, preparation, and MR acquisition procedures can be found in (25). An overview of the elephant specimen used in this study is provided in Table 1.

### Specimen Status

Specimen status varied widely in our study. Most heads reached us frozen, and none of the elephant heads/brains were perfused. Even though many of the included animals were dissected by professional veterinarians, material preservation varied largely across specimens. A few factors that contributed to suboptimal preservation of elephant material, included (but not limited to) the following: it often takes days to dissect elephants, elephant carcasses cool down very slowly; the freezing process causes peculiar artifacts; and the fixation process is quite slow even in extracted brains due to their size. Some of these problems following studies (5, 34).

### Methodological considerations of brain weight measurements in elephants

Determining elephant brain weights is more complex and prone to errors than brain weight estimates from other mammals. In addition, compiling brain weights from the literature poses problems. We briefly describe these problems here. Removing elephant brains and determining their weights is a complex several-hour process, particularly from adult animals with their pneumatized skulls. A typical problem is an incomplete removal or removal-related brain damage; for example, elephant olfactory bulbs are heavily attached to the olfactory nerve and often do not come out completely. We expect that incomplete removal will lead to small and varying underestimates of the actual brain weight. Another problem is the large pudding-like consistency of fresh brains, which are often not directly weighed. If weights are taken after a period of fixation, the fixative-induced shrinkage will also lead to small weight underestimates. Incomplete dura removal prior to weighing might lead to weight overestimates. As far as compiling brain weight data from the literature is concerned, the biggest problem is incomplete documentation. We often found studies with missing information like removal procedures, weighing protocols, and most importantly demographic data like sex and age. In the past, researchers (17) have tried to address these problems by excluding certain measurements, which they saw as unreasonable. We have not followed this example and instead included all values. Excluding “outlier” brains would lead to slightly lower brain weight estimates and more significant differences between groups, but would not affect any of our conclusions.

### Structural MR acquisition

The brains were stored in a 4% paraformaldehyde (PFA) solution prior to scanning. The brains were removed from their storage containers, drained of excess fluid, and placed in the head coil wrapped in a plastic bag filled with a phosphate-buffered solution, as a part of the scanning protocol.

MRI was performed on a Prisma 3T scanner (Siemens Healthcare, Erlangen, Germany) using a 20-channel head RF coil for signal reception. For structural analyses, a T1- and T2-weighted protocol was derived from the human connectome project (55) employing Siemens’ 3D product sequences (56) with voxel sizes scaled to the dimensions of elephant brains. Both structural scans were acquired with 1.1 mm isotropic resolution with a matrix size of 320 × 320 × 208 slices. Two-fold in-plane acceleration (GRAPPA) in the phase encode direction was used. For the T1-weighted scan, other parameters included: Repetition Time (TR) / Inversion Time (TI) = 2,500/1,000, Echo Time (TE) = 2.1 ms, flip angle of 8°. For the T2-weighted scan, other parameters included TR/TE = 3,200/564 ms, turbo factor = 314. All images were processed using the freely available open-source software ITK-SNAP (57) which allowed us to stitch and visualize the scanned volume.

As an outcome, we have high-resolution T1- and T2-weighted MRI volumes of brains from both babies and adults. Multicontrast structural MRI is very helpful in robust segmentation, especially as elephant brains are more than often damaged, and their brains’ MR scans are rarely acquired. The imaging quality and resolutions of these scans are excellent, with voxel volumes around 1.3 mL. We treasure this database and welcome the scientific community to use or collaborate.

### MRI artifacts

Automated segmentation could not be performed, given tissue quality and technical reasons, described as follows. As elephant brains are larger than human heads, they just fit inside the MR head coil, triggering intense bias fields; hence, we observe extremely diverse voxel values for the same tissue. Moreover, the brains were fixed for different amounts of time when MR was acquired, inhibiting PFA from penetrating throughout the brain volume. These fixation borders were apparent for Dumba and Stillborn baby brain scans, albeit only in T1-weighted images. Furthermore, some of the brains were perfectly fine when inspected from the surface but showed internal damage. Following these issues, we did not acquire adequate segmentation using the machine-learning-based automated segmentation tools, as they are designed specifically for the human brain, whose structure is inherently different from the elephant brain.

After experimentation with automated and manual segmentation, we designed a pipeline suitable for all of our brains to achieve the segmentation with minimal manual labor work. We first masked both hemispheres (separately) and the cerebellum for each brain, using Amira. Then, each hemisphere scan was corrected for bias field, using N4BiasFieldCorrection (58) from ANTs, with spline spacing ranging from 50 to 150 and different combinations of convergence (multiples of 50), suited for individual scans. Further, we chose an isometric or the closest to isotropic resolution volume, for each elephant brain, and remapped all other volumes to match this resolution, using mri_vol2vol from FreeSurfer (59).

### Cerebellum segmentation

Cerebellum segmentation was carried out in horizontal slices in MR scans, and later fine modifications were applied using coronal and sagittal planes. Note that brainstems were omitted from this segmentation, i.e. the cerebellum proportions were reported without brainstems. We then estimated cerebellum and hemisphere volumes simply by multiplying the number of voxels with single-voxel volume for respective brain parts. Then, the whole-brain volume was estimated as a sum of hemispheric and cerebellum volumes. Both cerebella and brain weights were estimated using independent mass densities reported by (60). Please refer to Table 1 to see which brains are included in this analysis.

### Cortical segmentation

The bias-field-corrected scans with homogeneous resolutions and sizes were then processed for automated segmentation of GM, WM, and Pial matter using either of the following tools:

- FAST FMRIB's Automated Segmentation Tool (61), by FSL: “FAST” labels individual voxels based on Gaussian clustering in multidimensional space (achieved with multiple volumes). We used this for any elephant brain without PFA-artifact.
- Segmentator (62): “Segmentator” detects boundaries between any two tissue regions based on their voxel gradient. It works without any prior information for any of these tissues; rather, it takes tissue voxel variation information from the user via a graphical interface.
- Image processing tools from MATLAB: Any hemispheres that could not be processed with either of the above tools were processed with MATLAB for automated segmentation derived as a combination of histogram adjustment, gradient detection in 2D/3D and/or thresholding.

These treatments provided partially (∼50% accurately) segmented label volumes that were later manually corrected using “recon edit” tool in FreeSurfer.

### Volume estimates and surface rendering

We estimated GM volume simply by multiplying number of voxels with the GM label (from the segmentation volume) with the single voxel volume of the respective MR image. The estimate reported here includes GM from the whole brain, i.e. both neocortical and subcortical GM.

We used WM–GM–Pial matter segmentation further to render 3D surfaces and measure cortical thickness with tools from FreeSurfer. We generated surfaces for individual tissues using mri_tessellate. The surfaces are in the form of a highly dense mesh of equilateral triangles. We smoothened these WM and pial surfaces using mris_smooth, with smoothing iterations varying from 1 to 5 depending on noise in label volumes; the command also provided us with curvature values for each node.

We measured cortical thickness as the normal Euclidean distance two surfaces, using

mris_diff  --min-dist [wm-srfc] [pial-srfc] 1 [th-file]

--min-dist: finds closest node to the normal from wm-surface

[wm-srfc] and [pial-srfc] are input surface files

“1”- option to set exact-flag

Lastly, surface inflation was performed using mris_inflate with default parameters.

## Supplementary Material

pgaf141_Supplementary_Data

## Data Availability

An additional MR scan dataset is available at DOI: 10.12751/g-node.mb72l6/ on the German Neuroinformatics Node (GIN).
