# Supplementary material for: Larger brains and relatively smaller cerebella in Asian elephants compared with African savanna elephants
Source: PNAS Nexus. 2025 May 20;4(5):pgaf141. doi: 10.1093/pnasnexus/pgaf141 (PMC12089752; doi:10.1093/pnasnexus/pgaf141)
Supplement: pgaf141_Supplementary_Data [file pgaf141_supplementary_data.docx]

Supplementary Information

for

Larger Brains and Relatively Smaller Cerebella in Asian

Compared to African Savanna Elephants

Malav Shah, Olivia Heise, Peter Buss, Lin-Mari de Klerk-Lorist, Stefan Hetzer, John-Dylan Haynes, Thomas Hildebrandt and Michael Brecht

Corresponding author: Michael Brecht

**Email:** [michael.brecht@bccn-berlin.de](mailto:michael.brecht@bccn-berlin.de)

**This file includes:**

Supplementary Figures S1 and S2

Supplementary Figure 1


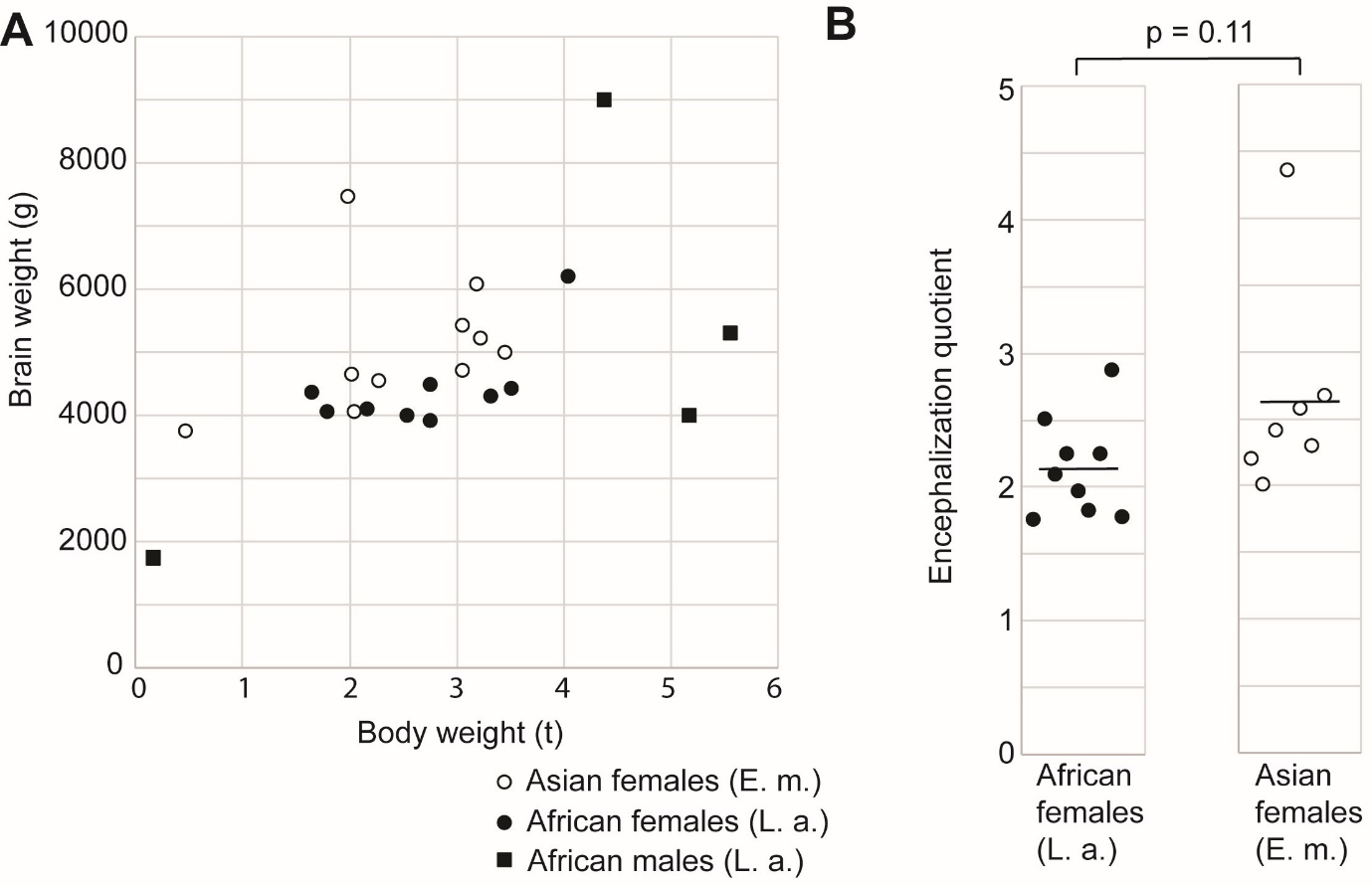


**Figure S1. Elephant body weight / brain weight plot and encephalization quotients (EQ)**

A) brain weight (in grams) plotted against body weight (in tons) for Asian (*Elephas maximus*) and African (*Loxodonta africana*) female and male elephants. Body and brain weight was available only for a limited number of animals. Note the higher brain weight of Asian female elephants for similar body weights

B) encephalization quotients (EQ) for adult African (*Loxodonta africana,* left) and adult Asian (*Elephas maximus,* right) female elephants. EQ were computed according to Jerison’s formula as applied in Shoshani et al 2006 (17); (brain weight in g) / ((body weight in g)^0.66 * 0.12). The number of entries is low and the values were not significantly different between African and Asian female elephants (unpaired t-test). Lines indicate means.

Supplementary Figure 2


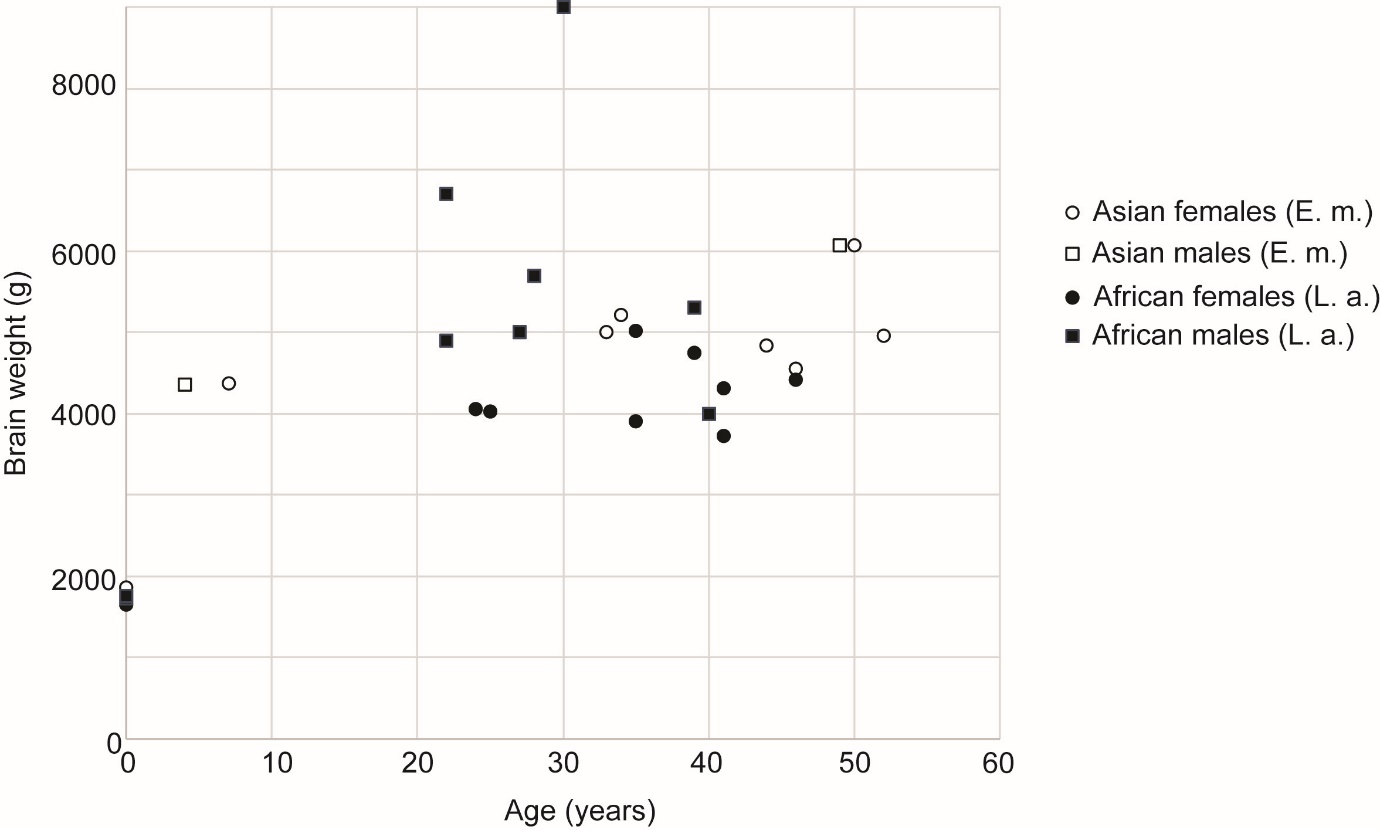


**Figure S2. Elephant brain weight vs age plot**

Brain weight (in grams) plotted against age (in years) for Asian (*Elephas maximus*) and African (*Loxodonta africana*) male and female elephants.
